# Lower Network Functional Connectivity Is Associated With Higher Regional Tau Burden Among Those At-Risk of Alzheimer’s Disease But Cognitively Unimpaired: Specific Patterns Based on Amyloid Status

**DOI:** 10.21203/rs.3.rs-5820051/v1

**Published:** 2025-01-20

**Authors:** Jamie Ford, Rosaleena Mohanty, Eric Westman, Lefkos Middleton

**Affiliations:** Imperial College London; Karolinska Institutet; Karolinska Institut; Imperial College London

**Keywords:** Alzheimer’s disease, Dementia, Resting-state, PET, MRI, Biomarkers, Amyloid, Tau, Functional Connectivity

## Abstract

**Introduction:**

Functional connectivity within the medial temporal lobe (MTL) and default mode network (DMN) changes across Alzheimer’s disease stages, influenced by and influencing cortical amyloid-beta (Aβ) and regional tau burden. Previous research highlights functional connectivity’s role in Alzheimer’s disease progression and the interactions of cortical Aβ and functional connectivity within and between the MTL and DMN, but their impact on regional tau deposition remains largely unexplored.

**Methods:**

Cognitively unimpaired participants from OASIS-3 (AV1451 cohort, *n*=287) were classified into Aβ− (*n*=193) and Aβ+ (*n*=94) groups via amyloid-PET for cross-sectional analyses. Principal components analysis of functional connectivity identified two MTL-functional connectivity and DMN-functional connectivity principal components (PCs), which were correlated with regional tau per Braak stages 1–6 brain regions. Aβ status-specific robust regressions evaluated whether functional connectivity was associated with tau.

**Results:**

In Aβ− participants, lower “MTL Integration Axis” functional connectivity (PC1) was associated with higher tau levels in the left entorhinal cortex. In Aβ+ participants, lower “MTL Integration Axis” functional connectivity correlated with elevated tau levels in the DMN’s left lateral parietal cortex, MTL’s right parahippocampal cortex, and Braak stages 3–6 brain regions.

**Discussion:**

Decreased functional connectivity was associated with increased regional tau burden, showing Aβ status-specific effects. Enhancing MTL functional connectivity could be a therapeutic strategy and a promising direction for future clinical interventions.

## Background

Alzheimer’s disease is a complex neurodegenerative disorder marked by a progressive decline in memory and functionality (Jack et al., 2024). Central to its pathology are hallmark pathological biomarkers: amyloid-beta (Aβ) plaques and neurofibrillary tau tangles (Jack et al., 2024). Aβ plaques form from the accumulation of Aβ peptides (Chen et al., 2017) and exhibit a less stereotypical distribution over time compared to the more systemic spread of tau pathology (Braak & Braak, 1991). Tau tangles follow a well-defined spatial pattern known as Braak staging, starting in the entorhinal cortex, progressing to the hippocampus and other limbic regions, and eventually spreading across the neocortex (Braak & Braak, 1991).

Functional connectivity refers to the statistical relationships, such as correlations, between different brain regions, reflecting the dynamic communication within the brain (Friston, 2011). Unlike structural connectivity, which maps the physical or anatomical connections between neural elements (Sporns, 2013), functional connectivity measures how the brain coordinates its activity over time (Friston, 2011). Functionally connected brain regions support cognitive and behavioural functions (Litwińczuk et al., 2022).

The Medial Temporal Lobe (MTL) encompasses crucial structures like the hippocampus, entorhinal cortex, and parahippocampal cortex, all of which are essential for memory formation, spatial information processing, and navigation (Squire et al., 2004). The Default Mode Network (DMN), first described by Raichle et al. (2001), includes brain regions such as the medial prefrontal cortex, posterior cingulate cortex, precuneus, and inferior parietal lobule (Raichle et al., 2001). These regions demonstrate synchronised activity during rest and introspective processes such as self-referential thinking, daydreaming, mind wandering, and recalling of personal experiences (Raichle et al., 2001; Greicius et al., 2002; Huntley et al., 2021).

Research indicates that elevated cerebral levels of Aβ and tau disrupt neural communication and connectivity across multiple brain networks (Vogel et al., 2020; Ingala et al., 2021). This disruption underlines the intricate relationship between molecular pathology and functional connectivity alterations in neurodegenerative diseases like Alzheimer’s disease. For instance, Giorgio et al. (2023) demonstrated that Aβ plaques, measured using positron emission tomography (PET), disrupt brain network interactions in cognitively unimpaired older adults during task-based functional magnetic resonance imaging (fMRI) activity. Specifically, Aβ flips connections between MTL and DMN from inhibitory to excitatory, leading to increased activity in the MTL, which predicted faster tau accumulation in the entorhinal cortex.

Despite technological advancements in brain imaging and the increasing popularity of functional connectivity in the field of Alzheimer’s disease research, significant knowledge gaps remain regarding the precise mechanisms linking Alzheimer’s disease biomarker accumulation and local functional connectivity patterns within brain networks and regions vulnerable to Alzheimer’s disease pathology (Aβ and tau), such as the MTL and DMN. The differential impact of these biomarkers on functional connectivity in various brain regions, particularly in cognitively unimpaired individuals, requires further investigation. Understanding these interactions may prove useful for developing targeted therapeutic strategies to potentially slow or prevent the progression of Alzheimer’s disease.

Hence, this study aimed to explore the relationships between MTL and DMN functional connectivity and regional tau burden in MTL and DMN brain regions, whilst stratifying participants into cortical Aβ risk groups (Aβ-negative [−] and Aβ-positive [+]). Previous studies have demonstrated that functional connectivity can predict tau distribution among cognitively unimpaired and Alzheimer’s disease participants (Franzmeier et al., 2019). Thus, we hypothesised that network-level functional connectivity would be associated with regional tau burden. Additionally, we anticipated that these relationships would differ between individuals with lower versus higher cortical Aβ burden. As prior research has indicated that elevated Aβ levels are linked to changes in both regional and global functional connectivity, even in individuals experiencing subjective cognitive decline (Li et al., 2021).

## Methods

### Study Design

The Open Access Series of Imaging Studies (OASIS) aims to provide free access to multimodal imaging datasets (LaMontagne et al., 2019). For this project, T1-structural MRI, resting-sate fMRI, Aβ-PET, tau-PET data were downloaded from the third OASIS study cohort (OASIS-3). This cross-sectional dataset includes participants who all underwent an ^18^F-AV1451 (AV1451) tau-PET scan, allowing for the analysis of the relationships between functional connectivity and tau pathology at a single time point. All brain imaging scans included in this current study occurred within six months of each other.

### Study Participants

OASIS-3 unimpaired participants (*n* = 287) with available T1-structural MRI, resting-state fMRI, Aβ-PET, AV1451 tau-PET data were further subdivided based on evidence of lower or elevated levels of cortical Aβ burden (Aβ− and Aβ + respectively). Among these participants, 193 exhibited lower cortical Aβ burden (Aβ−; 67.25%), while 94 showed evidence of higher cortical Aβ burden (Aβ+; 32.75%). Detailed inclusion and exclusion criteria for participant selection in the OASIS-3 study are provided in LaMontagne et al. (2019).

### Brain Imaging

#### Magnetic Resonance

The structural MRI data in the OASIS-3 study were acquired using 3 Tesla Siemens scanners with T1-weighted Magnetisation Prepared Rapid Gradient Echo sequences. Key parameters include a repetition time of approximately 2400 ms, an echo time of around 2.20–3.16 ms, and an inversion time of 1000 ms. The flip angle ranged between 8–10°, with a voxel size of 1 mm^3^ isotropic. The field of view was set at 256 mm, and the matrix size was 256 × 256.

For the resting-state fMRI data, the OASIS-3 study utilised Echo Planar Imaging sequences on 3 Tesla Siemens scanners. The repetition time for these scans was 2.20 seconds, with an echo time of 27 ms and a flip angle of 90°. The voxel size was set at 4 mm^3^ isotropic, and the field of view was 256 mm with a matrix size of 64 × 64.

#### Creating Anatomical Masks for Functional Connectivity Analyses

For this current study, Freesurfer (version 7.4.1) was utilised to create two additional anatomical masks not available in the CONN toolbox (Whitfield-Gabrieli et al., 2012). These masks delineated the left and right portions of the entorhinal cortex using the Desikan-Killiany atlas (Desikan et al., 2006). This process involved two key files: aparc.DKTatlas + aseg.mgz and a T1 structural image standardised in Montreal Neurological Institute space for reference.

**Figure 1:**
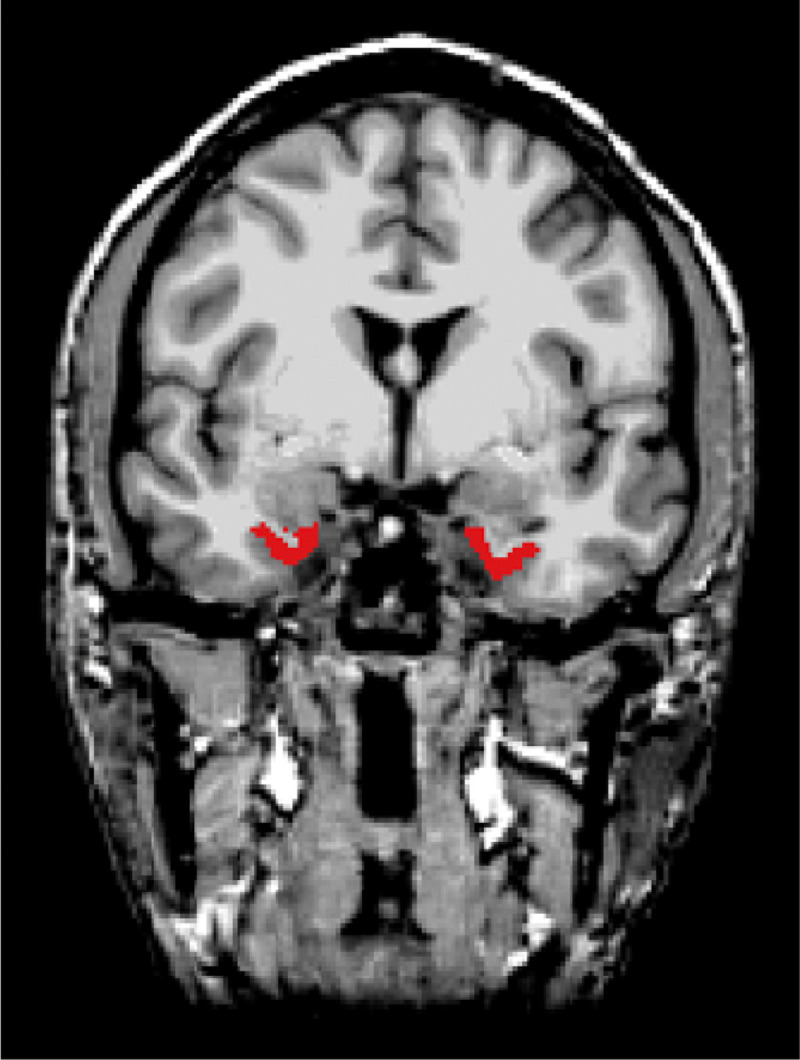
Anatomical masks of the left and right portions of the entorhinal cortex, shown in red. Both regions were created in Freesurfer (version 7.4.1).

The left and right portions of the entorhinal cortex were isolated as separate regions of interest using reference labels derived from the FreeSurferColorLUT.txt file (with label value 1006 for the left entorhinal cortex and 2006 for the right ERC). These regions of interest were visualised in FreeView (Fig. 1.). The .mgz files were subsequently imported into the CONN toolbox, and the brain regions were integrated into the Setup space within CONN’s graphical user interface. Following this integration, the data underwent denoising (see Pre-processing and Denoising section below) before being included as regions of interest in functional connectivity analyses.

#### Pre-processing and Denoising

Structural MRI and resting-state fMRI data were pre-processed using the CONN toolbox (version 22a). CONN’s default preprocessing pipeline was chosen. To summarise, first resting-state fMRI data was realigned using SPM12’s realign and unwarping procedure (Andersson et al., 2001). Structural MRI and resting-state fMRI scans were then co-registered and resampled to the first scan using *b*-spline interpolation. Next, slice time correction for temporal misalignments was made due to images being acquired in 2D slices over time, whereby fMRI data is time-shifted and resampled using *sinc*-interpolation to match the time in the middle of each acquisition time. Then, due to resting-state’s increased susceptibility to motion artifacts, CONN’s outlier detection step identifies outlier scans from the observed global blood oxygen level dependent signal, providing detailed estimates of the amount of participant motion using Artifact Detection Tool-based scrubbing. In this study, scans with framewise displacement > .9mm or global blood oxygen level dependent signal changes above five standard deviations (*SD*s) were considered as evidence of excessive motion. Functional and structural volumes were then segmented into separate tissue classes (grey matter, white matter, and cerebrospinal fluid), and then normalised into standard Montreal Neurological Institute space. Resting-state fMRI data were then smoothed using spatial convolution with a Gaussian kernel of 8mm full width half maximum to increase blood oxygen level dependent signal-to-noise.

Furthermore, due to resting-state’s susceptibility to motion and physiological-based artifacts, CONN also utilises a denoising pipeline that broadly includes two focus areas: (1) linear regression of potential confounding effects in the blood oxygen level dependent signal (e.g., “anatomical Component-based noise Correction” tool [Behzadi et al., 2007]), and (2) temporal band-pass filtering (.008 Hz or above .090 Hz).

#### PET

OASIS-3 participants included in this present study had both an amyloid- and tau-PET scan. Amyloid-PET scans were selected that were closest to their tau-PET. Amyloid-PET scans used one of two radiotracers: either Pittsburgh compound B (PiB) or ^18^F-AV-45 (florbetapir/AV45). 33.10% had a PiB amyloid-PET, and 66.90% had an AV45 amyloid-PET. Aβ status (−/+) was determined using two Aβ centiloid cut-offs, 16.40 and 20.60 for PiB and AV45, respectively. PET values were derived using a standardised protocol. PiB used a 30–60-minute post-injection window, AV45 used a 50–70-minute post-injection window, and the cerebellum cortex was used as the default reference region. See “*OASIS-3 Imaging Methods and Data Dictionary*” (OASIS-3, 2018; retrieved from: https://theunitedconsortium.com/wp-content/uploads/2021/07/OASIS-3_Imaging_Data_Dictionary_v1.8.pdf) for more information.

In the AV1451 sub-cohort of OASIS-3, participants also underwent AV1451 tau-PET scans, allowing for the assessment of regional tau burden within specific brain regions corresponding to Braak stages 1–6 (Braak & Braak, 1991). The brain regions associated with each Braak stage are as follows:

- **Braak stages 1–2**: Entorhinal cortex,
- **Braak stages 3–4**: Amygdala, Accumbens area, hippocampus, insula, medial orbitofrontal gyrus, lateral orbitofrontal gyrus, pars orbitalis, parahippocampal cortex, rostral anterior cingulate cortex, posterior cingulate cortex, caudal anterior cingulate cortex, and isthmus cingulate cortex, and,
- **Braak stages 5–6**: Banks of the superior temporal sulcus, middle temporal gyrus, caudal middle frontal gyrus, fusiform gyrus, inferior parietal gyrus, inferior temporal gyrus, lateral occipital gyrus, supramarginal gyrus, and precuneus.

For corresponding Freesurfer labels of these brain regions, see Table 1.

Additionally, as outlined in research by Chen et al. (2021), participants were assigned to Braak stages based on the severity of tau accumulation within Braak stages 1–6. This was based on the following criteria: participants with Braak 5/6 regions of interest standardised uptake value ratio (SUVr) > 1.873 were assigned as severity stage 4; participants with Braak 3/4 regions of interest SUVr > 1.523 were classified as severity stage 3; participants with Braak 3/4 regions of interest SUVr > 1.307 fell into severity stage 2; participants with Braak 1/2 regions of interest SUVr > 1.129 as severity stage 1; and lastly, participants with Braak 1/2 regions of interest SUVr ≤ 1.12 were assigned severity stage 0.

### Data Analysis

All brain imaging analyses were conducted using CONN (version 22a.). Functional connectivity was measured within and between the MTL and DMN via network-level region of interest-to-region of interest functional connectivity analysis.

**Figure 2:**
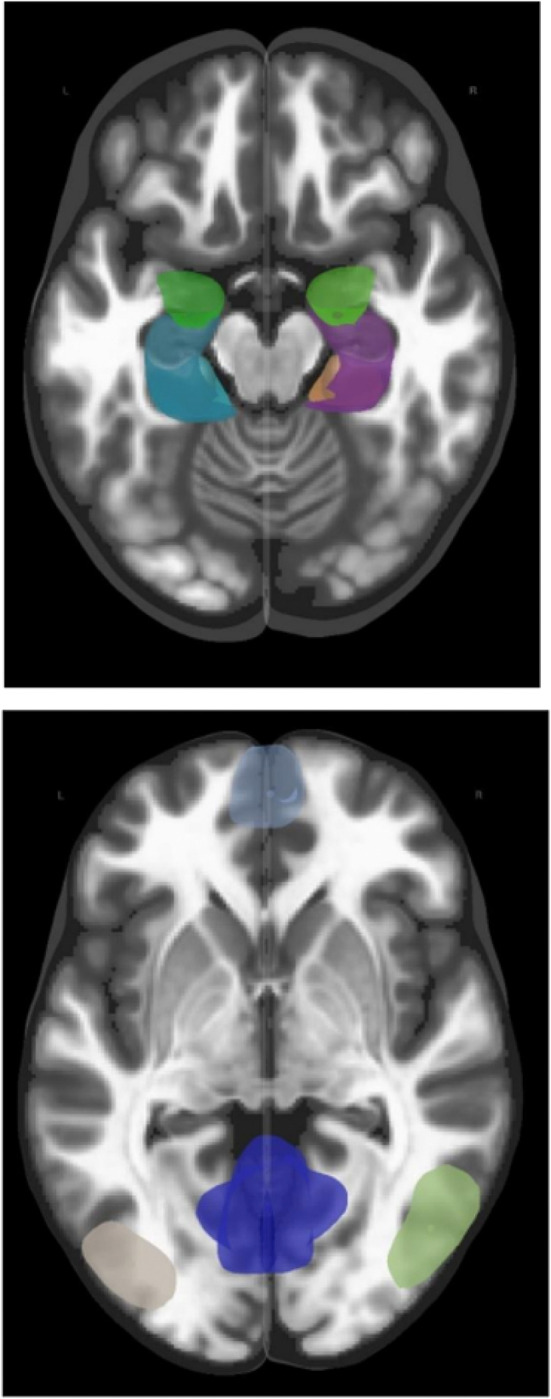
Top: brain regions comprising the Medial Temporal Lobe (MTL): left and right entorhinal cortex, left and right hippocampus, left and right portions of the anterior and posterior parahippocampal cortex, and left and right amygdala. Bottom: brain regions of the Default Mode network (DMN): medial prefrontal cortex, posterior cingulate cortex, and left and right parietal cortices. Red = the functional connections between brain regions.

The MTL consisted of the left and right entorhinal cortex, left and right hippocampus, left and right portions of the anterior and posterior parahippocampal cortex, and left and right amygdala (Squire et al., 2004); for a total of 10 regions (see Fig. 2.). The DMN consisted of the medial prefrontal cortex, posterior cingulate cortex, and left and right parietal cortices (Raichle et al., 2001); for a total of four regions (see Fig. 2.). As a result, across the whole imaging model, interconnections across 14 regions were measured (182 interconnections). Age, sex, and an adjusted total brain volume (TBV) measure were used as covariates of no interest.

${TBV}_{\text{adjusted}}$ was calculated in accordance with research by Voevodskaya et al. (2014), with the inclusion of estimated intracranial volume (ICV), whereby:

$${TBV}_{\text{adjusted}\;}=TBV-\beta (ICV-{ICV}_{\text{mean}})$$

Next, principal components analysis was used to reduce the dimensionality of functional connectivity data by transforming the data (small groups [or ‘clusters’] of functionally connected brain regions) into principal components (PCs), where each PC is orthogonal to the others and ordered by the amount of variance they explain (Groth et al., 2012). An oblique rotation was chosen to allow the PCs to be correlated (Dien, 2010), acknowledging the inherent relationships between brain regions. This approach reduced the number of connections down to their PCs, mitigating the possibility of making type I errors between functional connections and regional tau values.

Lastly, Spearman’s rank and robust regressions were used due to non-parametric data, as to explore the correlations between functional connectivity (independent variable) and regional tau (dependent variable), whilst stratifying by Aβ status.

**Figure 3:**
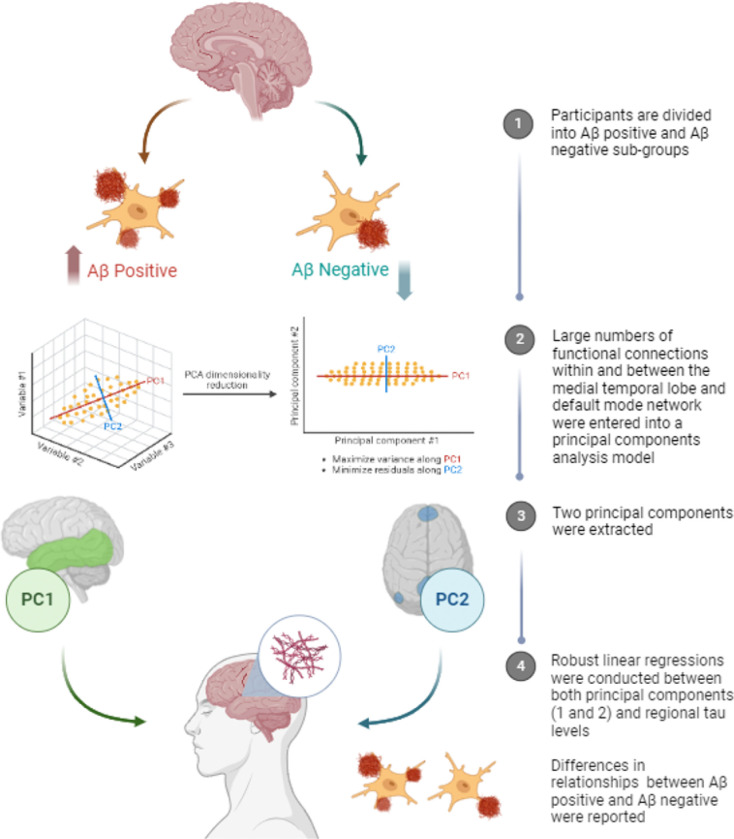
Conceptual illustration of analyses uses in this study. Aβ = amyloid-beta; PC1/2 = principal component 1/2.

A conceptual drawing for the data analyses used in this study can be found at Fig. 3.

## Results

### Demographics and Clinical Characteristics

In this study, participants with lower cortical Aβ burden (Aβ−) were, on average, younger (*M* = 66.70 years, *SD* = 8.26) compared to those with elevated Aβ burden (Aβ*+*; *M* = 72.10 years, *SD* = 6.63). The distribution of males and females was nearly equal across the entire sample (males = 41.90% [*n* = 122] and females = 58.10% [*n* = 169]) but differed notably when stratified by Aβ status. Specifically, the highest proportion of participants was female Aβ− (38.70% [*n* = 111]), whereas the lowest proportion was male Aβ+ (13.20%, *n* = 38).

Additionally, the mean TBV_adjusted_ values were equivalent for both Aβ− and Aβ + participants. However, Aβ + participants exhibited higher regional tau values and tau burden across all Braak stages. For a comprehensive overview of demographic and clinical characteristics, including stratification by Aβ status, see Table 2.

### Braak Staging

**Figure 4:**
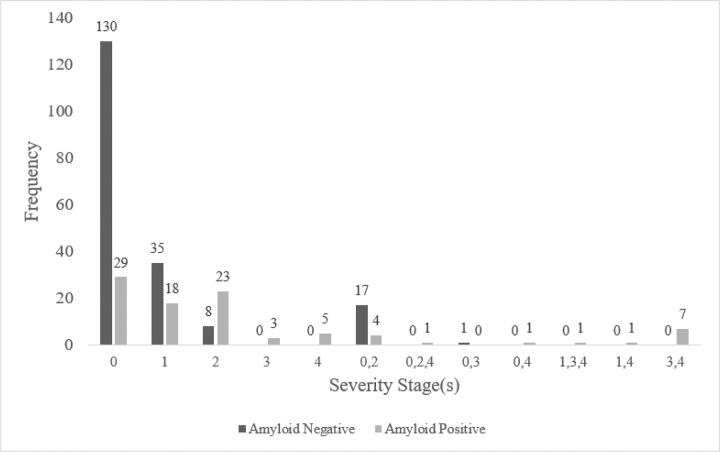
Frequency count. Participants were categorised in accordance with Chen et al.’s 2021 criteria: 0 = Braak stages I/II standardised uptake value ratio (SUVr) <1.129; 1 = Braak stages I/II SUVr between 1.129 and 1.307; 2 = Braak stages III/IV SUVr between 1.308 and 1.523; 3 = Braak stages III/IV SUVr between 1.524 and 1.873; and 4 = Braak stages V/VI SUVr >1.874. Braak stage regions in accordance with Braak and Braak (1991). Latter seven categories demonstrate overlap across multiple severity stages, e.g., “0,2” = Braak stages I/II SUVr <1.129 but Braak stages III/IV SUVr between 1.308 and 1.523.

Among the 193 Aβ− participants, 132 (68.39%) were classified as “0”; 35 (18.13%) as “1”; eight (4.15%) as “2”. No participants were classified as “3” or “4”. However, 18 (9.33%) participants were found to demonstrate evidence of atypical tau spread, e.g., showing overlap between multiple Braak stages and not fulfilling the criteria to be defined as a single stage. See also Fig. 4. Participants with overlapping criteria for multiple stages may suggest non-linear or atypical tau spread (e.g., Vogel et al., 2021).

In the Aβ + group of 94 participants, 30 (31.91%) were classified as “0”; 18 (19.15%) as “1”; 23 (24.47%) as “2”; three (3.19%) as “3”; five (5.32%) as “4”. 15 (15.96%) participants were found to demonstrate evidence of atypical tau spread (see also Fig. 4.).

### Region of Interest-to-Region of Interest Analysis

One hundred and sixty functional connections (of a possible 182) were statistically significant across 10 clusters (*p* < .05). These clusters were derived using optimal lead ordering for hierarchical clustering based on region of interest-to-region of interest anatomical proximity (see Bar-Joseph et al., 2001). The functional connections within each of the 10 clusters were averaged, forming 10 values (one per cluster) for each participant. This was conducted in this manner to reduce data dimensionality, minimise noise, and simplify the subsequent principal components analysis model, whilst attempting to maintain biologically plausible components.

The loadings of each cluster, as revealed by region of interest-to-region of interest analysis, were as follows: Cluster 1: “Within-functional connectivity” (left and right portions of the posterior parahippocampal cortex); Cluster 2: “Within-functional connectivity” (across DMN sub-regions); Cluster 3: “Within-functional connectivity” (across MTL sub-regions); Cluster 4: “Within-functional connectivity” (posterior parahippocampal cortex to other MTL sub-regions); Cluster 5: “Within-functional connectivity” (left and right portions of the entorhinal cortex); Cluster 6: “Between-functional connectivity” (MTL to DMN sub-regions [1]); Cluster 7: “Between-functional connectivity” (MTL to DMN sub-regions [2]); Cluster 8: “Within-functional connectivity” (entorhinal cortex to other MTL sub-regions); Cluster 9: “Within-functional connectivity” (entorhinal cortex to portions of the posterior parahippocampal cortex); and Cluster 10: “Between-functional connectivity” (entorhinal cortex to sub-regions of the DMN). Of the 160 total statistically significant connections (*p* < .05), 86 were “Within-MTL”, 12 were “within-DMN”, and 62 were “Between MTL-DMN”.

### Principal Components Analysis

Principal components analysis was conducted using the cluster averages 1–10 and revealing a two-factor solution (see Table 3.) which was validated visually using the scree plot. PC1 comprised Clusters 3, 4, 5, 8, and 9, while PC2 included Clusters 2, 4, 6, 7, and 10. Cluster 4 loaded significantly on both PC1 and PC2. Conversely, Cluster 1 did not load onto any PC until PC4. Given that the eigenvalue for a four-factor solution was < 1, Cluster 1 was excluded from subsequent analyses.

PC1 had a sum of squares value of 2.25 and explained 22.50% of the total variance, whilst PC2 had a sum of squares value of 2.15 and explained 21.50% of the total variance (with a cumulative variance of 44.00%). Inter-correlations between PC1 and PC2 were .23.

Bartlett’s test of sphericity was significant: *X*^2^(45) = 598, *p* < .001, and Kaiser-Meyer-Olkin measure of sampling adequacy (overall) = .67 and ranged from .44 (Cluster 1) and .83 (Cluster 2), supporting principal components analysis to be conducted with a two-factor solution. PCs1 and 2 were labelled based on the following rationales:

- **PC1: “MTL Integration Axis”**: this label denotes the integration of various connections within the MTL, including interactions between different structures within this region.
- **PC2: “DMN Integration Axis”**: similarly, this label conveys the integration of connections within the DMN, as well as its interactions with other brain regions, such as the MTL. It highlights the coordinated activity within the DMN and its communication with related brain regions

Shapiro-Wilk test was used to determine whether the distributions of PCs 1 and 2 followed a normal distribution. PC1 was not normally distributed (*W* = .98, *p* = .001) but PC2 was normally distributed (*W* = .99, *p* = .050).

### Spearman’s Rank Correlations Between Functional Connectivity and Tau Burden

#### In Cognitively Unimpaired Aβ− Individuals

One correlation was statistically significant between “MTL Integration Axis” functional connectivity and MTL, left entorhinal cortex, *ρ*=−.15, *p* = .038. There were no significant correlations observed between the other variables. Table 4. shows correlation strengths between “MTL Integration Axis” functional connectivity and “DMN Integration Axis” functional connectivity and tau burden of brain regions corresponding to Braak stages 1–6 among Aβ− participants.

#### In Cognitively Unimpaired Aβ + Individuals

Four relationships were statistically significant: “MTL Integration Axis” functional connectivity and DMN, left lateral parietal cortex tau, *ρ*=−.21, *p* = .047; and “MTL Integration Axis” functional connectivity and MTL, right parahippocampal cortex tau, *ρ*=−.22, *p* = .032. Furthermore, “MTL Integration Axis” functional connectivity and Braak stages 3–4 tau regions, *ρ*=−.30, *p* = .004; and “MTL Integration Axis” functional connectivity and Braak stages 5–6 tau regions, *ρ*=−.22, *p* = .033. Table 5. shows correlation strengths between “MTL Integration Axis” functional connectivity and “DMN Integration Axis” functional connectivity and tau burden of brain regions corresponding to Braak stages 1–6 among Aβ + participants

#### Robust Regression Models: Functional Connectivity Predicting Tau Burden

**Figure 5:**
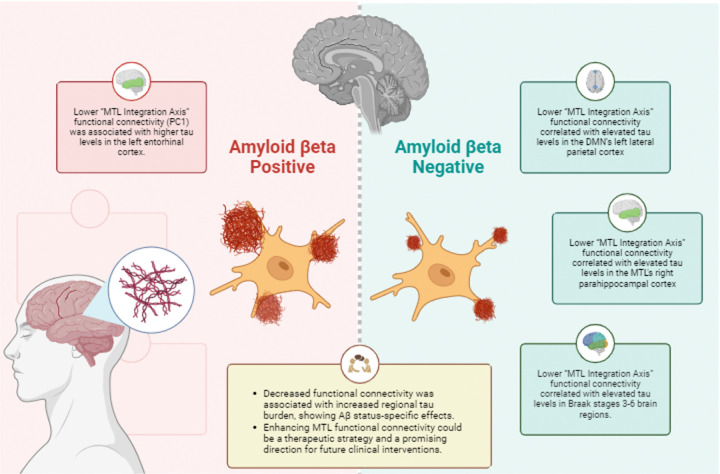
Visual summary of amyloid-beta (Aβ) status-specific results. MTL = Medial Temporal Lobe; DMN = Default Mode network; PC1 = principal component 1.

A visual summary of Aβ status-specific results can be found in Fig. 5.

#### In Cognitively Unimpaired Aβ− Individuals

One robust regression was statistically significant among Aβ− participants:

- **“MTL Integration Axis” Functional Connectivity predicting MTL, left entorhinal cortex tau**: the model fit statistics revealed an *R*^2^ value of .03 (explaining 2.50% of the variance in MTL, left entorhinal cortex tau), (*F*[1, 191] = 4.82, *p* = .029). ANOVA tests showed a significant model effect (sum of squares = .21, *F*[1, 191] = 4.82, *p* = .029), with “MTL Integration Axis” functional connectivity specifically contributing significantly (sum of squares = .21, *F*[1, 191] = 6.10, *p* = .014). The parameter estimates indicated that the intercept was .99 (*SE* = .02, *T*[191] = 66.53, *p* < .001), while “MTL Integration Axis” functional connectivity had a negative estimate of − .03 (*SE* = .01, *T*[191] =−2.47, *p* = .014), suggesting that as “MTL Integration Axis” functional connectivity increased, MTL, left entorhinal cortex tau decreased.

#### In Cognitively Unimpaired Aβ + Individuals

Four robust linear regressions were run for Aβ + participants (two for specific regional burden of tau and two across various Braak stages

- **“MTL Integration Axis” functional connectivity predicting DMN, left lateral parietal cortex tau**: the model fit statistics indicated an *R*^2^ of .05, meaning the model accounted for 5.00% of the variance in DMN, left lateral parietal cortex tau (*F*[1, 92] = 4.85, *p* = .030). ANOVA tests showed a significant effect for the model (sum of squares = 2.62, *F*[1, 92] = 4.85, *p* = .030, with “MTL Integration Axis” functional connectivity specifically contributing significantly (sum of squares = 2.62, *F*[1, 92] = 4.61, *p* = .034). Parameter estimates revealed the intercept at 1.65 (*SE* = .08, *T*[92] = 21.61, *p* < .001) and “MTL Integration Axis” functional connectivity with a negative estimate of − .17 (*SE* = .08, *T*[92]=−2.15, *p* = .034). Higher values of “MTL Integration Axis” functional connectivity are associated with lower values of DMN, left lateral parietal cortex tau.
- **“MTL Integration Axis” functional connectivity predicting MTL, right parahippocampal cortex tau**: the model fit statistics indicated an *R*^2^ of .07, meaning the model accounted for 7.40% of the variance in MTL, right parahippocampal cortex tau, (*F*[1, 92] = 7.33, *p* = .008). ANOVA tests showed a significant effect for the model (sum of squares = .71, *F*[1, 92] = 7.33, *p* = .008), with “MTL Integration Axis” functional connectivity specifically contributing significantly (sum of squares = .71, *F*[1, 92] = 7.23, *p* = .009). Parameter estimates revealed the intercept at 1.19 (*SE* = .032, *T*[92] = 36.94, *p* < .001) and “MTL Integration Axis” functional connectivity with a negative estimate of − .09 (*SE* = .03, *T*[92]=−2.69, *p* = .009), suggesting that higher values of “MTL Integration Axis” functional connectivity are associated with lower values of MTL, right parahippocampal cortex tau.
- **“MTL Integration Axis” functional connectivity predicting Braak stages 3–4 tau**: model fit statistics indicated an *R*^2^ of .09, meaning the model accounted for 8.80% of the variance in Braak stages 3–4 tau (*F*[1, 92] = 8.85, *p* = .004). ANOVA tests showed a significant effect for the model (sum of squares = .46, *F*[1, 92] = 8.85, *p* = .004) and specifically for “MTL Integration Axis” functional connectivity (sum of squares = .46, *F*[1, 92] = 5.34, *p* = .023). Parameter estimates revealed the intercept at 1.35 (*SE* = .02, *T*[92] = 56.74, *p* < .001) and “MTL Integration Axis” functional connectivity with a negative estimate of − .07 (SE = .03, t[92]=−2.31, *p* = .023), suggesting that higher values of “MTL Integration Axis” functional connectivity are associated with lower values of Braak stages 3–4.
- **“MTL Integration Axis” functional connectivity predicting Braak stages 5–6 tau**: model fit statistics indicated an *R*^2^ of .048, meaning the model accounted for 4.80% of the variance in Braak stages 5–6 tau (*F*[1, 92] = 4.68, *p* = .033). ANOVA tests showed a significant effect for the model (sum of squares = 1.47, *F*[1, 92] = 4.68, *p* = .033) and specifically for “MTL Integration Axis” functional connectivity (sum of squares = 1.47, *F*[1, 92] = 4.64, *p* = .034). Parameter estimates revealed the intercept at 1.56 (*SE* = .06, *T*[92] = 26.65, *p* < .001) and “MTL Integration Axis” functional connectivity with a negative estimate of − .13 (*SE* = .06, *T*[92]=−2.16, *p* = .034). Higher values of “MTL Integration Axis” functional connectivity are associated with lower values of Braak stages 5–6 tau.

## Discussion

This study aimed to explore the relationships between MTL- and DMN-functional connectivity and regional tau burden in both Aβ− and Aβ + cognitively unimpaired older adults. Our findings revealed that lower/impaired “MTL Integration Axis” functional connectivity is associated with higher regional tau. Notably, these relationships were more pronounced in individuals with elevated cortical Aβ deposition, underscoring the modulatory impact of Aβ pathology on tau distribution and network dynamics.

### Differential Impact of “MTL Integration Axis” Functional Connectivity and “DMN Integration Axis” Functional Connectivity on Tau Pathology Between Aβ Statuses

“MTL Integration Axis” functional connectivity (PC1) was positively correlated with “DMN Integration Axis” functional connectivity (PC2) in both Aβ− and Aβ + groups, with a significantly stronger correlation observed in the Aβ + participants. This stronger correlation might reflect Aβ-related hyperactivity, as higher levels of Aβ deposition are linked to increased activity within the MTL (Pasquini et al., 2019). This hyperactivity could enhance functional connectivity between the MTL and DMN, possibly due to Aβ-related accumulation and resulting network disruption.

The influence of “MTL Integration Axis” functional connectivity on tau pathology demonstrated variability across different Aβ status groups. Specifically, “MTL Integration Axis” functional connectivity was identified as a negative predictor of MTL, left entorhinal cortex tau levels, suggesting that, among those with lower levels of Aβ pathology (Aβ−; as measured by PET), higher “MTL Integration Axis” functional connectivity was associated with reduced tau burden within the left entorhinal cortex. However, the statistical significance of this relationship was accompanied by a modest *R*^2^ value, indicating that additional factors may also play a role in shaping this association.

Compared to Aβ− participants, those with elevated cortical Aβ levels exhibited more spatially varied and statistically significant relationships between “MTL Integration Axis” functional connectivity and regional tau burden. Although the *R*^2^ values were relatively modest, ranging from 4.80–8.80%, these findings suggest that “MTL Integration Axis” functional connectivity plays a more substantial role in predicting regional tau distribution in participants with elevated cortical Aβ levels. As Alzheimer’s disease is a highly heterogenous condition. So, elucidating additional sources of variance in functional connectivity-tau relationships may help uncover the pathophysiological processes occurring during the early stages of Alzheimer’s disease among older adults.

Functional connectivity along the “MTL Integration Axis” correlated with tau pathology in several regions, whereas functional connectivity along the “DMN Integration Axis” did not show significant correlations with tau pathology within any of the included regions. In AD, tau pathology tends to follow a stereotypical pattern, starting in the MTL and spreading to other cortical areas, as described by Braak staging (Braak & Braak, 1991). In our sample of cognitively unimpaired older adults, the lack of significant relationships between “DMN Integration Axis” functional connectivity and regional tau might reflect an earlier stage of tau spread. These relationships may become more apparent in later stages of the disease.

### Potential Mechanisms

In this cross-sectional study, the observation of increased functional connectivity alongside elevated cortical Aβ burden (measured by PET) may point towards early neurodegeneration. Increasing tau pathology is associated with the disruption of functional connectivity (Nabizadeh, 2024). As tau pathology increases, it exacerbates the loss of functional connectivity, which is critical for cognitive processes (Berron et al., 2021). Our results suggest a particular vulnerability of those with elevated cortical Aβ burden – despite no evidence of cognitive impairment - in moderating the relationships between functional connectivity and tau. The presence of higher cortical Aβ appears to disrupt these relationships within clinically pertinent brain regions. Namely, the relationships between “MTL Integration Axis” functional connectivity and tau within the DMN, left lateral parietal cortex; MTL, right parahippocampal cortex; and Braak stages 3–6 brain regions. This early neurodegenerative process, evidenced by the association between lower functional connectivity and higher tau pathology, highlights the impact of cortical Aβ on brain regions associated with cognition, even before clinical symptoms of impairment become apparent.

Higher “MTL Integration Axis” functional connectivity may also indicate stronger network integrity or adaptive neuroplasticity, potentially mitigating against the neurotoxic effects of tau accumulation. This interpretation is supported by previous research emphasising the crucial role of neuroplasticity in brain health. Synaptic plasticity - the ability of synapses to strengthen or weaken over time in response to activity changes - is essential for maintaining cognitive functions and promoting recovery after neural damage (Bassi et al., 2019). However, neuroplasticity diminishes with age, particularly in brain regions critical for learning and memory, such as the hippocampus (Wong et al., 2021).

Because of this, individuals with higher “MTL Integration Axis” functional connectivity may demonstrate cognitive resilience, maintaining cognitive functions despite the presence of tau pathology. We propose that enhancing functional connectivity and promoting neuroplasticity could serve as strategies to potentially mitigate against cognitive decline in older adults at-risk of Alzheimer’s disease.

One such strategy is utilising Transcranial Magnetic Stimulation, a non-invasive neuromodulation technique that modulates brain activity via a series of magnetic pulses to specific areas of the brain (Weiler et al., 2019). These pulses can influence neuronal excitability and synaptic plasticity (Weiler et al., 2019). By targeting regions outlined in this study, Transcranial Magnetic Stimulation (or similar imaging modalities) may enhance functional connectivity between these areas. This enhancement may aid in counteracting the functional connectivity and tau pathology changes observed during early stages of Alzheimer’s disease.

Another important concept closely related to neuroplasticity is cognitive reserve. Cognitive reserve is defined as the brain’s capacity to utilise its accumulated lifetime resources, such as education, intellectual engagement, and social activities, to mitigate the determinantal effects of brain pathology and sustain cognitive function (Stern, 2009). Recent research identified education as a significant determinant of both cognitive and brain resilience against tau pathology (Bocancea et al., 2023). In our study, higher “MTL Integration Axis” functional connectivity may reflect stronger network integrity. Therefore, it is hypothesised that these individuals exhibiting higher network integrity also possess greater cognitive reserve. By accounting for individual differences in cognitive reserve, we may enhance the explanatory power and applicability of our findings within general linear models predicting the relationships between functional connectivity and tau pathology.

Education has often been used as a rough proxy of cognitive reserve (Tucker & Stern, 2011). Surprisingly, Aβ + participants had slightly higher educational attainment compared to Aβ−. However, this difference was slight and data describing both years of education and Aβ status was only available for 51.57% of the total sample, and only 37 of which were Aβ + . Hence, to explore this hypothesis further, larger samples describing educational attainment, as well as other measures of cognitive reserve (e.g., occupational complexity, engagement in cognitive and social activities, and other measures) are needed.

Prior research by Giorgio et al. (2023), using task-based fMRI and multimodal PET imaging, revealed that Aβ deposition disrupts the excitatory-inhibitory balance in the DMN, resulting in hyperexcitation within the DMN itself. This hyperexcitation subsequently increased activity within the MTL and elevated MTL activity, driven by DMN hyperexcitation, was found to predict accelerated tau accumulation in the entorhinal cortex. These findings suggest that disturbances induced by Aβ in DMN function may initiate a cascade of neural changes contributing to the progression of tau pathology in Alzheimer’s disease. Our study aligns with Giorgio et al. (2023) in demonstrating Aβ’s impact on functional connectivity. However, unlike Giorgio et al. (2023), our study found that higher Aβ burden appears to alter the relationship between MTL functional connectivity and regional tau accumulation within the MTL itself, rather than the DMN. These differences may stem from methodological choices, such as the use of task-based fMRI versus resting-state fMRI. Furthermore, the complex interplay between Aβ, tau, and functional connectivity may exhibit non-linear effects, leading to differences in study outcomes.

### Clinical Implications and Potential Biomarkers

The results from this study may point towards clinical interventions aimed at increasing “MTL Integration Axis” functional connectivity to potentially modify tau pathology progression, even at the earliest stages of Alzheimer’s disease. By gaining further insight into the relationships between functional connectivity and tau progression, this may aid in informing disease models and therapeutic strategies.

In this cross-sectional study, “MTL Integration Axis” functional connectivity demonstrated significant relationships between regional tau burden across multiple regions, particularly when cortical Aβ were elevated. Therefore, functional connectivity could serve as a potential biomarker for early detection or progression of AD, particularly in the context of cortical Aβ pathology.

Stam et al. (2023) explored the relationship between excitation-inhibition balance, which involves the dynamic equilibrium of neural signals, and functional connectivity. Previous research by Schoonhoven et al. (2022) characterised Alzheimer’s disease by alterations in functional connectivity across different frequency bands – specifically, a decrease in functional connectivity in higher frequency bands and an increase in functional connectivity in lower frequency bands. Building on this, Stam et al. (2023) suggested that the excitation-inhibition balance is modulated by two factors: the connection strength and the frequency band.

This understanding aligns with studies demonstrating network hyperexcitability in Alzheimer’s disease animal models, associated with abnormal protein accumulation that may exacerbate further deposition of these abnormal proteins (e.g., Tombini et al., 2021). These insights underscore the potential of functional connectivity as a potential biomarker for Alzheimer’s disease, offering valuable implications for early diagnosis and therapeutic interventions.

The finding that most cognitively unimpaired participants were in early Braak stages (0 or 1), but some were progressing to later stages (2–4), highlights the importance of early detection and ongoing monitoring. Furthermore, the observation of atypical tau accumulation patterns that do not align with traditional Braak staging suggests that Alzheimer’s disease may not always follow a predictable path (Vogel et al., 2021). There is also a need for more flexible and comprehensive assessment tools that consider individual variability in regional pathological accumulation, as well as a need to further explore the mechanisms underlying this atypicality.

### Strengths and Limitations

#### Strengths

This study enriches our understanding of the complex and intricate relationships between functional connectivity, Aβ deposition, and tau accumulation in a cohort of cognitively unimoaired older adults. It sheds light on how differences in Aβ deposition across groups may impact the associations between MTL and DMN functional connectivity and tau burden within the same brain regions.

Furthermore, this study employed principal components analysis as a method to mitigate the risk of type 1 errors. This statistical method was utilised to enhance the robustness of the findings by effectively managing high data dimensionality of functional connections within and between the MTL and DMN.

Additionally, the research assessed regional tau accumulation in brain areas known for their vulnerability in early Alzheimer’s disease stages, specifically the MTL and DMN (Huijbers et al., 2019; Gardini et al., 2021), and incorporated groups of brain regions targeted by tau pathology and how it spreads in accordance with Braak staging.

### Limitations

This study is not without limitations. Firstly, as mentioned previously, the cross-sectional study design precludes tracking the relationships between functional connectivity and pathology over time. As Aβ and tau burden increases, new brain regions may emerge as statistically significant, and already observed relationships may change.

Lastly, despite this study’s strengths in incorporating measures of regional tau, and the OASIS-3 study’s approaches to undergo rigorous preprocessing, analysis, and quality control procedures to ensure reliability and validity, one cannot rule out the possible confounding of off-target binding in structures such as the hippocampus (Baker et al., 2017; Lemoine et al., 2018). Off-target binding can confound results by leading to inaccuracies in quantifying the true extent of tau pathology (Passamonti et al., 2017). Consequently, caution needs to be exercised when interpreting PET data with off-targeting binding effects.

### Future Research

Future research would benefit from conducting longitudinal studies to observe how the relationships between functional connectivity and tau levels evolve over time. Additionally, interventional trials targeting these relationships, such as those aiming to enhance functional connectivity through cognitive training or neuromodulation techniques, could provide insights into whether reducing tau burden leads to improved cognitive and/or functionality outcomes. These studies may also advance our understanding of personalised medicine by identifying specific interventions that are most effective for individual patients based on their unique neural (network) functional connectivity patterns and (both cortical [Aβ] and regional [tau]) pathological profiles. Gaining further insight into the relationships between functional connectivity and tau may aid in informing Alzheimer’s disease models and early therapeutic strategies in the future.

## Conclusions

This study emphasises the significance of local, network-level, functional connectivity modulating regional tau deposition, contingent on Aβ status. Specifically, among participants with lower Aβ levels, lower “MTL Integration Axis” functional connectivity was associated with higher tau levels in the left entorhinal cortex. Whereas, among participants with higher Aβ levels, lower “MTL Integration Axis” functional connectivity correlated with elevated tau levels in the DMN’s left lateral parietal cortex, MTL’s right parahippocampal cortex, and Braak stages 3–6 brain regions.

The findings suggest that enhancing functional connectivity of the medial temporal lobe could serve as a potential therapeutic target, offering a promising avenue for future clinical interventions aimed at mitigating tau-related neurodegeneration in AD, particularly among those with elevated cortical Aβ.

## Figures and Tables

**Table 1:** Brain Regions Corresponding to Each Braak Stage and Their Freesurfer Label Names

Braak Stage	Freesurfer Region Processing Name	Brain Region
*1–2*	ctx-entorhinal	Entorhinal cortex
*3–4*	Amygdala	Amygdala
	Accumbens-area	Accumbens area
	Hippocampus	Hippocampus
	ctx-insula	Insula
	ctx-medialorbitofrontal	Medial orbitofrontal cortex
	ctx-lateralorbitofrontal	Lateral orbitofrontal cortex
	ctx-parsorbitalis	Pars orbitalis
	ctx-parahippocampal	Parahippocampal cortex
	ctx-rostralanteriorcingulate	Rostral anterior cingulate cortex
	ctx-posteriorcingulate	Posterior cingulate cortex
	ctx-caudalanteriorcingulate	Causal anterior cingulate cortex
	ctx-isthmuscingulate	Isthmus cingulate
*5–6*	ctx-bankssts	Banks of the temporal sulcus
	ctx-middletemporal	Middle temporal gyrus
	ctx-caudalmiddlefrontal	Caudal middle frontal gyrus
	ctx-fusiform	Fusiform gyrus
	ctx-inferiorparietal	Inferior parietal gyrus
	ctx-inferiortemporal	Inferior temporal gyrus
	ctx-lateraloccipital	Lateral occipital gyrus
	ctx-supramarginal	Supramarginal gyrus
	ctx-precuneus	Precuneus

**Notes.** ctx = cortex.

**Table 2.** Demographic and Clinical Characteristics of Aβ- and Aβ+ Participants

Variable		Aβ Status	
	Whole Sample (*n*=287)	Aβ− (*n*=193; 67.25%)	Aβ+ (*n*=94; 37.75%)

**Demographics**			

***Age***			
(M | *SD*)	68.50 (*8.16*)	66.70 (*8.26*)	72.10 (*6.63*)

***Sex*** (%)			
*Males*	122 (*41.90*)	82 (*28.69*)	38 (*13.20*)
*Females*	169 (*58.10*)	111 (*38.70*)	56 (*19.50*)

***Years of Education^1^***	148 / 287 (*51.57*)	111 / 148 (*75.00*)	37 / 148 (*25.00*)
(*M | SD*)	16.32 (*2.29*)	16.30 (*2.26*)	16.41 (*2.41*)

**APOE**			

***APOE Carrier Status** (%)* ^2^			
*Non-Carriers*	88 / 146 (*60.27*)	77 / 146 (*52.74*)	11 / 146 (*7.53*)
*Carriers*	58 / 146 (*39.73*)	33 / 146 (*22.60*)	25 / 146 (*17.12*)

**Brain Outcomes**			

***Structural MRI***			

TBV_adjusted*_ (mm^3^)			
(M | *SD*)	1.50e^+6^ (*181697*)	1.50e^+6^ (*178413*)	1.50e^+6^ (*187501*)

***Tau-PET***			

MTL, Left Entorhinal Cortex** (SUVr)			
(M | *SD*)	1.12 (*.38*)	.99 (*.21*)**	1.39 (*.51*)

***MTL, Right Entorhinal Cortex*** (SUVr)			
(M | *SD*)	1.14 (*.38*)	1.02 (*.26*)	1.38 (*.48*)

***MTL, Left Hippocampus*** (SUVr)			
(M | SD)	1.33 (*.24*)	1.28 (*.22*)	1.45 (*.23*)

***MTL, Right Hippocampus*** (SUVr)			
(M | *SD*)	1.32 (*.23*)	1.26 (*.21*)	1.44 (*.22*)

***MTL, Left Parahippocampal Cortex***(SUVr)			
(*M*| *SD*)	1.06 (*.25*)	.99 (*.16*)	1.21 (*.33*)

MTL, Right Parahippocampal Cortex** (SUVr)			
(M | *SD*)	1.05 (*.26*)	.98 (*.18*)	**1.19 (*.32*)** **

***MTL, Left Amygdala*** (SUVr)			
(M | *SD*)	1.25 (*.38*)	1.12 (*.19*)	1.52 (*.50*)

***MTL, Right Amygdala*** (SUVr)			
(M | *SD*)	1.27 (*.40*)	1.13 (*.19*)	1.57 (*.54*)

***DMN, Medial Prefrontal Cortex*** (SUVr)			
(M | *SD*)	1.05 (*.26*)	.99 (*.17*)	1.16 (*.37*)

***DMN, Posterior Cingulate Cortex*** (SUVr)			
(M | *SD*)	1.37 (*.31*)	1.30 (*.16*)	1.51 (*.45*)

***DMN, Left Lateral Parietal Cortex***(SUVr)			
(M | *SD*)	1.39 (*.49*)	1.26 (*.18*)	1.65 (*.75*)

***DMN Right Lateral Parietal Cortex*** (SUVr)			
(M | *SD*)	1.39 (*.46*)	1.27 (*.16*)	1.63 (*.71*)

***Braak Stages 1–2 Tau*** (SUVr)			
(M | *SD*)	1.13 (*.37*)	1.01 (*.21*)	1.38 (*.47*)

Braak Stages 3–4 Tau** (SUVr)			
(M | *SD*)	1.23 (*.19*)	1.18 (*.12*)	**1.35 (*.24*)** **

Braak Stages 5–6 Tau** (SUVr)			
(M | *SD*)	1.34 (*.37*)	1.24 (*.12*)	**1.56 (*.57*)** **

***Notes:*** Aβ=amyloid status (−/+); M=mean; SD=standard deviation; SUVr = standardised uptake value ratio; TBV_adjusted_ =an adjusted measure of total brain volume; **MTL=medial temporal lobe**;

**DMN=default mode network;. Braak stages 1–2:** entorhinal cortex, **Braak stages 3–4:** amygdala, Accumbens area, hippocampus, insula, medial orbito-frontal gyrus, lateral orbito-frontal, pars orbitalis, parahippocampal cortex, rostral anterior cingulate cortex, posterior cingulate cortex, caudal anterior cingulate cortex, isthmus cingulate cortex, **Braak stages 5–6:** banks of the superior temporal sulcus, middle temporal gyrus, caudal middle frontal gyrus, fusiform gyrus, inferior parietal gyrus, inferior temporal gyrus, lateral occipital gyrus, supramarginal gyrus, and precuneus.

* TBV_adjusted_ was calculated in accordance with prior work by Voevodskaya et al. (2014).

** Functional connectivity was found to be associated with tau levels within this region.

**Table 3.** Component Loadings for Cluster Averages 1–10

	Principal Component		

	PCI	PC2	Uniqueness

*C1* *	-	-	.97
*C2*	-	.61	.50
*C3*	.61	-	.49
*C4*	.56	.33	.49
*C5*	.56	-	.71
*C6*	-	.85	.30
*C7*	-	.83	.32
*C8*	.84	-	.33
*C9*	.63	-	.61
*C10*	-	33	.89

***PC Summaries***	C3, C4, C5, C8, and C9	C2, C4, C6, C7, and C10	

Notes. Component loadings for cluster averages 1–10. PC = principal component; C=cluster. Underlined=cross-loadings across PCs 1 and 2. C1: “Within-functional connectivity” (left and right portions of the posterior parahippocampal cortex); C2: “Within-functional connectivity” (across default mode network [DMN] sub-regions); C3: “Within-functional connectivity” (across medial temporal lobe [MTL] sub-regions); C4: “Within-functional connectivity” (posterior parahippocampal cortex to other MTL sub-regions); C5: “Within-functional connectivity” (left and right portions of the entorhinal cortex); C6: “Between-functional connectivity” (MTL to DMN sub-regions [1]); C7: “Between-functional connectivity” (MTL to DMN sub-regions [2]); C8: “Within-functional connectivity” (entorhinal cortex to other MTL subregions); C9: “Within-functional connectivity” (entorhinal cortex to portions of the posterior parahippocampal cortex); and C10: “Between-functional connectivity” (entorhinal cortex to sub-regions of the DMN).

* C1 did not load onto any PC until PC4, but the eigenvalue was <1.

**Table 4.** Correlation Matrix Showing Relationships Between Functional Connectivity and Braak Stage Tau Burden Among **Aβ−**

		“MTL Integration Axis” Functional Connectivity	“DMN Integration Axis” Functional Connectivity	Braak (1–2)	Braak (3–4)	Braak (5–6)

*“MTL Integration Axis” Functional Connectivity*						
	*df*					
	*p-value*					
						
*“DMN Integration Axis” Functional Connectivity*		.18**				
	*df*	191				
	*p-value*	**.013**				
					
*Braak (1–2)*		.00	−.14			
	*df*	191	191			
	*p-value*	.984	.058			
				
*Braak (3–4)*		−.09	.03	.26***		
	*df*	191	191	191		
	*p-value*	.637	.636	**<.001**		
			
*Braak (5–6)*		−.03	.01	.25***	.67***	
	*df*	191	191	191	191	
	*p-value*	.637	.872	**<.001**	**<.001**	

Note.

* *p*<.05,

** *p*<.01,

*** *p*<.001, Aβ−=amyloid-beta negative participants; MTL = medial temporal lobe; DMN = default mode network;; *df*=degrees of freedom. Warmer colours denote the strength of positive relationships; cooler colours denote the strengths of negative relationships. Braak stages 1–2: entorhinal cortex, Braak stages 3–4: amygdala, Accumbens area, hippocampus, insula, medial orbito-frontal gyrus, lateral orbito-frontal, pars orbitalis, parahippocampal cortex, rostral anterior cingulate cortex, posterior cingulate cortex, caudal anterior cingulate cortex, isthmus cingulate cortex, Braak stages 5–6: banks of the superior temporal sulcus, middle temporal gyrus, caudal middle frontal gyrus, fusiform gyrus, inferior parietal gyrus, inferior temporal gyrus, lateral occipital gyrus, supramarginal gyrus, and precuneus.

**Table 5.** Correlation Matrix Showing Relationships Between Functional Connectivity and Braak Stage Tau Burden Among **Aβ+**

		“MTL Integration Axis” Functional Connectivity	“DMN Integration Axis” Functional Connectivity	Braak (1–2)	Braak (3–4)	Braak (5–6)

*“MTL Integration Axis” Functional Connectivity*						
	*df*					
	*p-value*					
					
*“DMN Integration Axis” Functional Connectivity*		.77***				
	*df*	92				
	*p-value*	**<.001**				
					
*Braak (1–2)*		−.11	.02			
	*df*	92	92			
	*p-value*	.279	.840			
				
*Braak (3–4)*		−.30**	−.17	.61***		
	*df*	92	92	92		
	*p-value*	**.004**	.095	**<.001**		
			
*Braak (5–6)*		−.22*	−.17	.37***	.78***	
	*df*	92	92	92	92	
	*p-value*	**.033**	.105	**<.001**	**<.001**	

Note.

* *p*<.05,

** *p*<.01,

*** *p*<.001. Aβ−=amyloid-beta negative participants; MTL = medial temporal lobe; DMN = default mode network;; *df*=degrees of freedom. Warmer colours denote the strength of positive relationships; cooler colours denote the strengths of negative relationships. Braak stages 1–2: entorhinal cortex. Braak stages 3–4: amygdala, Accumbens area, hippocampus, insula, medial orbito-frontal gyrus, lateral orbito-frontal, pars orbitalis, parahippocampal cortex, rostral anterior cingulate cortex, posterior cingulate cortex, caudal anterior cingulate cortex, isthmus cingulate cortex. Braak stages 5–6: banks of the superior temporal sulcus, middle temporal gyrus, caudal middle frontal gyrus, fusiform gyrus, inferior parietal gyrus, inferior temporal gyrus, lateral occipital gyrus, supramarginal gyrus, and precuneus.
